# Dry matter losses and quality changes during short rotation coppice willow storage in chip or rod form

**DOI:** 10.1016/j.biombioe.2018.02.005

**Published:** 2018-05

**Authors:** Carly Whittaker, Nicola E. Yates, Stephen J. Powers, Tom Misselbrook, Ian Shield

**Affiliations:** a Department of Agro-Ecology, Rothamsted Research, Harpenden, Hertfordshire, AL5 2JQ, UK; b Department of Computational and Systems Biology, Rothamsted Research, Harpenden, Hertfordshire, AL5 2JQ, UK; c Department of Sustainable Soils and Grassland Systems, Rothamsted Research, North Wyke, Okehampton, Devon, EX20 2SB, UK

**Keywords:** Biomass, Fuel quality, Storage, Supply chain

## Abstract

This study compares dry matter losses and quality changes during the storage of SRC willow as chips and as rods. A wood chip stack consisting of approximately 74 tonnes of fresh biomass, or 31 tonnes dry matter (DM) was built after harvesting in the spring. Three weeks later, four smaller stacks of rods with an average weight of 0.8 tonnes, or 0.4 tonnes DM were built. During the course of the experiment temperature recorders placed in the stacks found that the wood chip pile reached 60 °C within 10 days of construction, but the piles of rods remained mostly at ambient temperatures. Dry matter losses were calculated by using pre-weighed independent samples within the stacks and by weighing the whole stack before and after storage. After 6 months the wood chip stack showed a DM loss of between 19.8 and 22.6%, and mean losses of 23.1% were measured from the 17 independent samples. In comparison, the rod stacks showed an average stack DM loss of between 0 and 9%, and between 1.4% and 10.6% loss from the independent samples. Analysis of the stored material suggests that storing willow in small piles of rods produces a higher quality fuel in terms of lower moisture and ash content; however, it has a higher fine content compared to storage in chip form. Therefore, according to the two storage methods tested here, there may be a compromise between maximising the net dry matter yield from SRC willow and the final fine content of the fuel.

## Highlights

• Willow cut as wood chips and rods were stored in stacks for 6 months.
• The wood chip stack lost 18.9–23.5% of the DM compared to 0–9% lost in the rods.
• Storing willow as rods produces a lower moisture and ash content fuel.
• Chipped rods have a higher fine content compared to stored chips.

## 1 Introduction

One of the most challenging aspects of using biomass for energy is preserving dry matter and fuel quality during storage [1]. Due to the limited harvesting window of short rotation coppice (SRC) willow, the crop must be stored between harvesting in late winter/early spring and eventual consumption by a bioenergy facility. Willow is typically harvested at just over 50% moisture content (MC), so it is beneficial to dry and store the material simultaneously in order to provide a suitable quality fuel at the time of demand [1]. Two studies have shown that dry matter (DM) losses of short rotation coppice willow and poplar are around 20% [2,3], when storing in stacks for between three and 9 months, respectively, though periodical sampling in Ref. [3] showed that the DM losses plateaued after four to five months. The wood chip stacks showed rapid increases in temperature to around 60 °C within a few days of establishment, with a corresponding increase in CO_2_ concentration within them. The DM losses were found to be higher than in studies on forest-residue chips, which may be due to the higher proportion of bark in short rotation woody chips. Bark contains many plant nutrients and, after comminution, offers an ideal growth medium for bacteria and fungi [4].

The initial heating phase is suggested to create favourable conditions for microbial and fungal colonisation. The transfer of heat and moisture between the wood chip stack and the outside air is dependent on the equilibrium relationship between them and the rate at which the moisture can diffuse through the stack [5]. Stacks consisting of larger particles should follow ambient temperatures more closely than those formed of smaller particles where self-heating is prevalent [[5], [6], [7], [8]]. Willow can be harvested as chips, as billets (∼20 cm pieces) and as whole stems (rods). In the UK, chipping is most commonly carried out, due to a number of modified forage harvesters being available, and this offers multiple use of harvest machinery. There is currently one active billet harvester and no more than two rod harvesters in operation in the UK. There have been no studies examining the DM losses of SRC willow when stored as rods in the UK, although it is hypothesised that such storage will reduce dry matter losses and allow the biomass to dry more effectively due to natural ventilation in the stacks [9]. Unfortunately, rod harvesting increases costs because the material is more difficult to handle than chips and because another processing stage in the supply chain is required, as the rods must be chipped before being combusted. This study explores the DM losses and quality of wood chips produced from SRC willow when stored as chips in a large pile, or as rods in smaller piles, which are later chipped.

Dry matter losses can lead to complications, however, as other characteristics may change during storage that affects the combustion properties of the fuel. For example, ash contents have been shown to increase during wood chip storage due to decay of the biodegradable fractions [1]. Also, the washing away of water-soluble components, such as salts and alkali chlorides, by rain can change characteristics of the ash such as ash fusibility [10]. Ash content can also increase due to contamination from soil or from dust particles in wind [11]. Moreover, natural composting processes, can alter the carbon-to-nitrogen ratio (C:N) during storage [12]. This often leads to a higher relative fuel-bound nitrogen composition of the biomass which causes higher emissions of NO_x_ during combustion [13]. Finally, changes in the quantities of fines (particles less than 3.15 mm) during storage must be assessed, as these tend to burn rapidly and generate very high temperatures in combustion systems. This can lead to ash melting and slagging [14]. Fines can also have important health and safety implications for those handling biomass [15]. Another aim of this study is to explore quality changes during wood chip and rod storage.

## 2 Materials and methods

### 2.1 Wood chip stack construction

The material for the wood chip storage pile was harvested from two areas of SRC willow. The sites were established in 2009 and were previously harvested in winter 2011–12. The areas were planted with breeding material from a *S. viminalis x S. schwerinii* cross. Both sites were previously cropped in an arable rotation. The SRC willow was treated with a residual herbicide (aminotriazole) and 60 kg ha^−1^ nitrogen in spring 2012 to encourage re-growth.

**Fig. 1:**
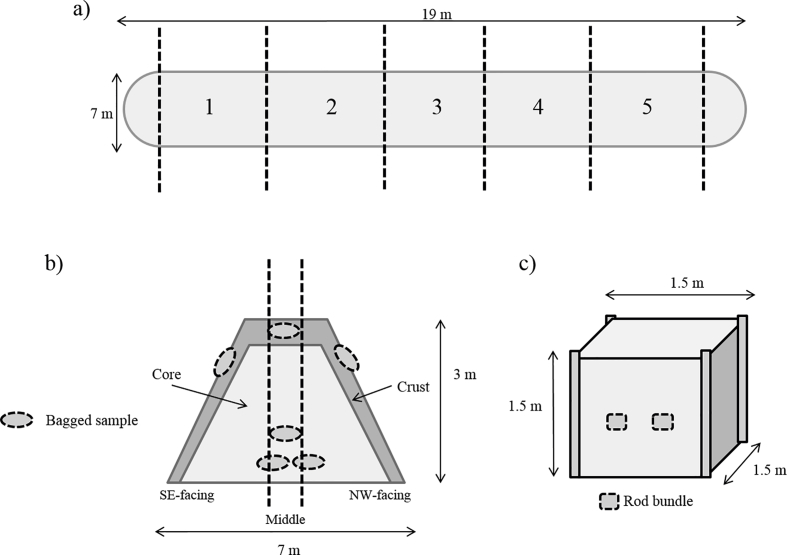
Diagrams of experimental set ups of wood chip and rod stacks. A) shows a birds-eye view of the wood chip stack with sample sections identified, b) shows a side profile of the wood chip stack with location of plastic mesh bags in the crust, core and middle of the stack and c) shows the typical structure of the rod stacks and location of the rod bundles.

The crop was harvested on the 4th March 2015 using a Claas forager harvester with a Coppice Resources Ltd (Retford, UK) header and blown into an accompanying trailer. At this point ten fresh samples of wood chips were taken for MC, ash and composition analysis. The material was immediately transported to a nearby field (coordinates 52.012854, −0.598906) where the stack was built by tipping the chips onto the ground and piling them up using a tractor with a front mounted loader and bucket. The completed stack was approximately 19 m long, 7 m wide and 3 m high and was built in a precise south-westerly to north-easterly orientation (Fig. 1).

The total stack mass was determined by weighing the harvested material during the stack establishment. This was performed by manoeuvring the trailer over a series of portable weight pads (PT Weigh pads, Weightru, Stourbridge, UK) on a concrete standing. Each pad can weigh up to 10,000 kg to the nearest 100 kg. The weight under each axle was recorded, as well as under the drawbar. The readings from the weight pads were validated by comparing the weight of the empty trailers with their known weights from a calibrated weighbridge.

### 2.2 Rod stack construction

The material for the rod storage stacks was taken from a similar plant breeders trial site, where the two experiments consisted mainly of pure *Salix viminalis* genotypes. Both experiments were situated approximately 40 km from that giving rise to the wood chip. The first experiment was harvested on 4th February 2014 and the second experiment was harvested on 30^th^ March 2015. Both were weighed onsite in batches and placed adjacent to the harvesting site (coordinates 51.812,413, −0.375,903) where one (2014 experiment) and four (2015 experiment) stacks were built. Both the harvesting and building of the piles was performed by hand to mimic the action of a machine such as the Stemster III (Nordic Biomass, Denmark). At this point four samples of rods were chipped and taken for MC, ash and composition analysis. These samples were chipped by an arboricultural chipper, not by the forage harvester used for the chip storage. Each stack was supported within an area pre-marked with 1.5 m-high wooden stakes approximately 1.5 m apart in a square formation (Fig. 1c), and the rods were placed between the stakes to make the stack. In 2014 the single stack contained 1212 kg of fresh biomass (567 kg DM), whereas in 2015 the stacks contained an average of 763 kg of fresh biomass (358 kg DM), both somewhat less that the 4000 kg that can be accommodated on the bed of a Stemster machine prior to tipping the rods to make a stack.

### 2.3 Dry matter loss assessment

Dry matter losses were assessed in both the wood chip and rod storage piles by drying weighed samples at 80 °C for 4 days to deduce the change in mass. All reported MCs are on a wet basis. A previous study found a great deal of heterogeneity in terms of DM losses and MCs within two wood chip stacks, therefore a strategic method of assessment was employed to attempt to reduce uncertainty in the results. The same study also found that a ‘crust’ developed on the outer layer of the stack, therefore the current study attempted to examine losses occurring in this layer. For this, the stack was divided into five zones (Fig. 1a). In each zone the dry matter losses occurring in the core, outer layer and top of the stack were tested using weighed, independent plastic mesh bag samples of approximately 3–4 kg of the freshly harvested wood chips. As the pile was being built, three bags were placed in the core area of each zone so that they were at least 2–3 m under the surface (Fig. 1b). In each zone, one bag was placed in the top of the stack. Finally, in each zone, two bags were placed on either side of the outer layer of the stack, about 2 m high, and buried so that they were flush with the outer surface. A temperature recorder (Log Tag^®^ Model Trix-8, LogTag Recorders Ltd. Auckland, New Zealand) was added to each core bag to log the temperature on a two-hourly basis throughout the storage period.

In the rod stacks, dry matter losses were measured using small independent bundles (approx. 20 kg) made from randomly selected stems, which were tied together and weighed. In 2014, eight bundles were placed within the central region of the stack, whereas, in 2015, three bundles were placed in the central region of each rod stack, as it was being built. In 2015, a temperature recorder was placed in the centre of each stack.

### 2.4 Stack breakdown

The wood chip stack was dismantled after 208 days, on 28th September 2015 using a tractor with a front mounted loader. During breakdown, care was taken not to lift soil with the loader bucket so that a bed of chip remained on the ground that was no more than 15 cm in depth. However, this depth ranged across the site and in some instances small amounts of soil were lifted. The lifted chip was loaded onto a series of trailers which were re-weighed on the same concrete hard standing with the portable weigh pads.

The bagged samples were retrieved, and the moisture and ash content was determined for these samples. Due to previously observed heterogeneity in wood chip piles, some extra samples were taken. From each zone, two (technical replicate) samples were taken for moisture content analysis from the crust and the core of the south-eastern (SE) and north-western (NW) sides of the stack, and also from the ‘middle’ of the profile (Fig. 1b). One sample from each technical replicate was taken for ash analysis. Ten random samples of the stack after lifting and re-tipping were taken to assess the overall moisture content change during the experiment, and a large sample was taken to analyse the particle size.

In 2014, the collected chip was then weighed, giving some indication of losses during the handling and chipping process. The rod stacks were dismantled after 156 and 192 days, on 10th July 2014 and 8th October 2015 respectively. The work was conducted by hand, without collecting finer debris from the bed of the stack. The whole stacks were weighed and then chipped. A total of ten mixed samples of the chip for each original stack were taken for analysis.

### 2.5 Fuel quality assessment

The following analyses were performed for the wood chip and rod stack experiments carried out in 2015. Samples were dried at 80 °C for 72 h to derive the MC. For ash determination, dried samples were ground using a hammer mill to provide material that would pass through a 1 mm mesh. The ash component of the biomass was determined after drying the milled biomass for 12 h at 105 °C then heating in a muffle furnace to 550 °C for 6 h according to the National Renewable Energy Laboratory (NREL) methodology [16]. The total carbon and nitrogen composition of the samples were determined using a LECO CN628 combustion analyser (LECO, Stockport, UK), based on a modified version of the Dumas digestion method.

Particle size distribution was assessed based on CEN/TS 15,149–1. Firstly, the wood chip was dried at 80 °C for 72 h to enable an easy flow between the particle size sieves. Five large sub-samples of dried chip were weighed and filtered through a series of sieves with aperture sizes 100 mm, 63 mm, 45 mm, 16 mm and 3.15 mm. The series of sieves was placed on a flat-bed shaker until all particles had ceased moving. The material remaining on each sieve, including the material that had fallen through the 3.15 mm sieve, was then weighed. The particle size distribution was then calculated as a percentage for each particle size class based on the total mixed sample.

The bulk density of the chip was estimated using dried material. The chip was placed into a container of a known volume (2600 cm^3^) until it was full and level with the top. The sample was weighed and bulk density was calculated. This procedure was done for five random samples of dried material to provide an average value.

### 2.6 Statistical analysis

Due to the lack of replication in the wood chip stack, the results from the wood chip and rod stacks are not statistically compared. Instead, changes in moisture content, ash, C, N and particle size distributions from pre- and post-storage samples were analysed using Student's t-test to assess the effect of storage for both wood forms.

The extra MC and ash data from the wood chip stack were analysed using a linear mixed model to test (F-tests) for the main effects and interaction between locations (SE, NW, middle) and regions (core, crust) having taken account of the nesting of regions within locations within zones. Data on percentage moisture and ash content and dry matter loss using the bagged samples were modelled similarly, to compare the crust to the core of the stack, taking account of bags within regions within zones. After all linear mixed modelling, appropriate predicted means and standard error of the difference (SED) values for the comparison of means were output.

The GenStat (17th edition, ^©^ VSN International Ltd, Hemel Hempstead, UK) statistical package was used for all analyses.

## 3 Results

### 3.1 Temperature profile

**Fig. 2:**
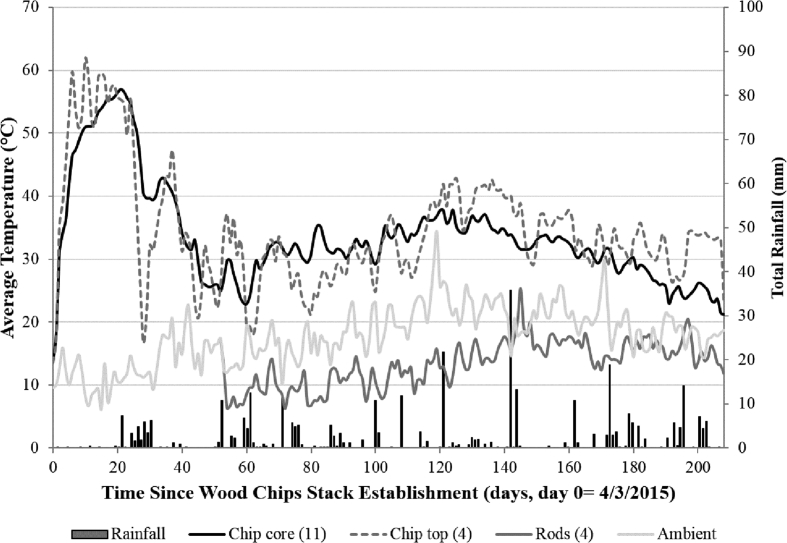
Average daily temperatures from temperature recorders placed in the top and core of the wood chip stack, and in the centre of the rod stacks (2015), alongside ambient temperatures and daily sums of rainfall (mm).

Different temperature profiles were seen in the wood chip and rod stacks. Fig. 2 shows the average of records retrieved from the Log Tag^®^ recorders placed in the biomass stacks, alongside rainfall (for the wood chip site). Rainfall and ambient temperatures were taken from daily meteorological records from weather stations located at each site. In 2015 the ambient maximum temperatures did not substantially differ between the two sites between March and October, with average maximum temperatures of 17 and 17.8 °C at the chip and rod sites, respectively. While the chip and rod experiments were being carried out they each received 354 and 412 mm of rainfall, respectively. In 2014, the average maximum temperature between February and July was 15.3 °C and total rainfall was 257 mm.

Overall, 19 Log Tag^®^ recorders were retrieved from the wood chip stack: 11 from the core of the stack and four from the top, but the other four were damaged. The recorders showed that the top of the wood chip stack reached 62 °C ten days after stack establishment, and that the core warmed at a slower pace, reaching 58 °C after 20 days. The core temperature of the stack remained at over 50 °C until day 28, and afterwards cooled to around 30 °C. In general, the top of the stack was warmer than the core until day 70 when a crossover occurred. However, after 120 days (during the summer months) the top was once again most usually warmer than the core. The trend was similar to that observed in previous experiments on SRC willow [2,17] and poplar [3,7].

All four temperature recorders were retrieved from the rod stacks and indicated that there had been no self-heating. Apart from on the first two days, when the temperature of the rod stacks was the same as ambient temperature, the rod stacks remained cooler than ambient levels. Interestingly, on 27th July (day 145, Fig. 2) ambient temperature decreased greatly after a major rainfall event, but the stacks were about 6 °C warmer. However, this only lasted for 2 days until ambient levels increased again.

### 3.2 Moisture contents and dry matter losses

#### 3.2.1 Wood chip stack

**Fig. 3:**
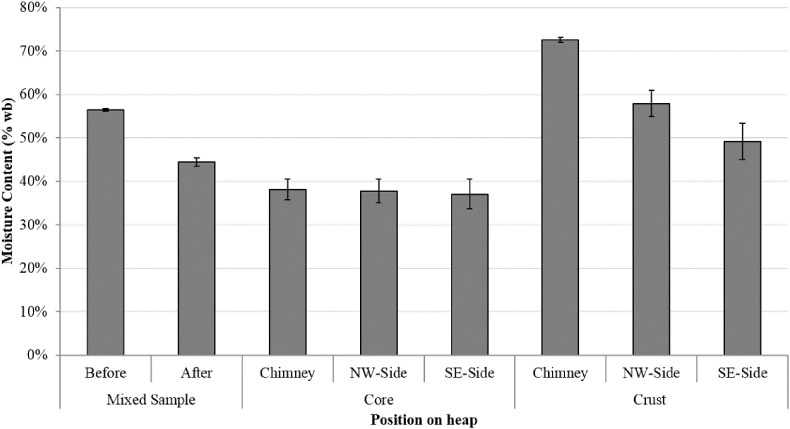
Moisture content of samples taken from different sides of the wood chip stack averaged across the five sample sections. Error bars show standard error.

The freshly harvested wood chip had an average MC of 56.4% (SE 0.5%) and the stack was built with approximately 73,480 kg of chip, which was weighed by combining 21 separate trailer loads. Considering the margin of error on the weight pads used, this corresponds to between 71,380 and 77,580 kg, with an equivalent dry matter content of between 31,096 and 32,926 kg. The MC of the chip from the core was relatively consistent over the zones (average 38.2%, SE 1.4) and was significantly drier than the outer crust, which averaged 59%, SE 2.4 (F = 91.20 on 1 and 42 df, p < 0.001, Fig. 3). The crust showed large variations in MC and varied in depth throughout the stack, with sporadic larger damp ‘hot spots’, and/or the presence of white mould. The top of the stack was the wettest part (MC 72.4%), indicating the same ‘chimney effect’ observed in other studies of stacked biomass, with transition of water upwards where it cools and condenses [1,[18], [19], [20]]. Hence, there was a significant interaction between location (NW-side, SE-side, middle) and region (crust, core) (F = 9.77 on 2 and 42 df, p < 0.001). The crust of the SE-side was drier (49.2%) than that of the NW side (57.9%), suggesting some effect of solar radiation.

At the end of the experiment, mixed samples of the wood chip had an average MC of 44.4% (Table 1), but ranged between 39.9 and 50.4% (SE 1.8%). The total stack weight was 45,410 kg, measured using 18 separate measurements (trailer loads), the range being between 43,610 and 47,210 kg, when accounting for the margin of error in the method of weighing. Based on the standard error of recorded MC in the stored chip, this corresponds to a total dry matter loss of 23,790 and 26,701 kg in the final pile, or a whole stack loss of between 18.9 and 23.5%. This includes losses due to decay and due to the loss of chip at the bottom of the heap, which could be described as a ‘handling’ loss.

**Table 1:** Details of parameters of samples of chip from the wood chip storage experiment before and after storage.

Parameter	Pre-storage %	Post storage %	SED	*t*-test	t-value	d.f
Moisture content	56.4^a^	44^a^	1.04	p < 0.001	11.52	9
Ash content	1.7	2.5	0.23	p = 0.011	3.7	5.6
C content	49.0	49.8	0.81	p = 0.36	1.01	5.1
N content	0.3	0.5	0.02	p < 0.001	6.34	10
*Particle size*						
16-45 mm	15.5	6.7	1.64	p < 0.001	5.71	8
3.15–16 mm	78.4	89.2	1.90	p < 0.001	5.45	8
<3.15 mm	5.7	4.1	1.35	p = 0.39	0.96	4.1
Bulk density	173	144	2.8	p < 0.001	9.77	8

a Wet basis, all other measurements stated as dry basis.

Three of the core bags were damaged when the pile was lifted and could not be used for DM loss assessment. All of the plastic mesh bags that were positioned in the NE and SW crust of the stack were disturbed and ripped opened by animals. The remaining 17 plastic mesh bag samples showed a grand mean DM loss of 23.1% (SE 3.3%). There was no difference in DM loss between bags found in the core and at the top of the stack (F = 0.91 on 1 and 8 df, p = 0.368). However, as found for the non-bagged samples, bagged samples at the top had a marginally higher (F = 16.05 on 1 and 4 df, p = 0.016) and more variable average MC (59.4%, SE 8.6%) than those found in the core of the stack (average 26.4%, SE 1.7%).

#### 3.2.2 Rod stacks

As the rods were harvested at different times to the wood chips, the freshly harvested and chipped samples of SRC rods had a slightly lower average MC of 53.2% in 2014 and 53.1% in 2015, but this was more variable (SE 1.5 and 1.8%, respectively). At the end of the experiment, mixed samples of the chipped rods had a significantly (p < 0.001, *t*-test) lower MC of 21.8% (SE 1.2%) and 23.9% (SE 0.8%) for the 2014 and 2015 stacks, respectively, compared to the original material. The rod pile in 2014 was stored for a slightly shorter duration (6 months) compared to those in 2015 (8 months), yet dried slightly better, which may be due to the lower rainfall during the course of the experiment in 2014. The overall moisture content in the stored rod chips was less variable than seen in the wood chip study.

**Fig. 4:**
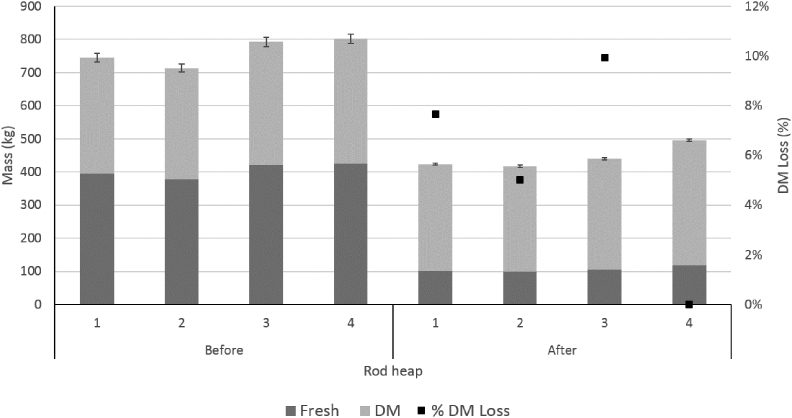
Fresh and dry matter content of the four rod stacks before and after storage (2015), with the estimated corresponding dry matter (DM) losses, based on the whole stack (see section 3.2.2).

In 2014, the single stack of rods lost an average of 1.9% (SE 1.4%) of the dry matter present at harvest. In 2015, the four rod stacks lost 5.9% (SE 2.6%) and 4.3% (SE 1.8%) of the DM present at harvest based upon the weight of the whole stacks and the weight of the bundled rods contained within, respectively (Fig. 4). The large variance (0–9.9%) in the estimated DM losses across all rod heaps may be due to the high variation in starting moisture contents of the freshly harvested rods.

### 3.3 Ash changes during storage

There was no noticeable difference in ash contents in the rod samples before and after storage (Table 2). In the wood chip study, there was a marginal increase in ash content after storage, increasing from an average of 1.7%–2.5% (Table 1). One sample containing 11.1% ash was excluded as this was obviously heavily contaminated with soil. When comparing the pre-storage material with the specific post-storage samples taken from the NW, SW and ‘middle’ post-storage, there was also an increase in ash, however this was not statistically significant (p > 0.05, t-tests). Further analysis of post-storage material from the combinations of location and region showed higher ash contents in the crust (2.0%) compared to the core (1.8%), but this was not statistically significant (F = 1.25 on 1 and 12 df, p = 0.286). Finally, when considering the bagged samples, there was a marginally significant difference in the ash content in the core (1.7%) compared to the crust (2.0%, F = 6.65 on 1 and 15 df, p = 0.024).

**Table 2:** Details of parameters of samples of chip from chipped rods before and after storage for the four rod stacks in 2015.

Parameter	Pre-storage %	Post storage %	SED	*t*-test	t-value	d.f
Moisture content	53.1^a^	23.9^a^	0.017	p < 0.001	17.31	9
Ash content	1.6	1.5	0.0008	0.39	0.89	10
C content	50.1	49.5	0.0034	0.12	1.77	7.6
N content	0.3	0.4	0.0002	0.14	1.59	10
*Particle size*						
16-45 mm	6.7	6.2	2.78	0.36	0.97	8
3.15–16 mm	87.5	82.5	21.35	0.22	1.32	8
<3.15 mm	5.3	11.1	2.89	0.003	4.23	8
Bulk density	184	179	5.65	0.394	0.9	8

a Wet basis, all other measurements stated as dry basis.

### 3.4 Carbon and nitrogen composition changes

In the wood chip stack, there was a significant (p < 0.001, *t*-test) increase in the nitrogen (N) content of the stored material (0.45%) compared to the fresh material (mean 0.31%, Table 1), and as the overall carbon (C) content did not change this resulted in a lower C:N ratio in the stored material. The rod chip samples showed no statistically significant (p > 0.05, *t*-test) change in either C or N contents.

### 3.5 Particle size distribution changes

There were some notable changes in particle size over the storage period for both the wood chips and rods. In wood chips there was a 10% increase in the smaller, 3.15–16 mm, fraction after storage (Table 1), which corresponded to the decrease in larger, 16–45 mm, particles seen after storage. However, the amount of fines, or particles smaller than 3.15 mm, did not significantly (p = 0.390, *t*-test) change. There were no particles larger than 45 mm in any of the samples. This result was also associated with a significant (p < 0.001, *t*-test) decrease in the bulk density of dried chip after storage from an average, taken over 5 samples, of 173 kg/m^3^ to 144 kg/m^3^, showing that the stored wood chip consisted of smaller, lighter and less dense pieces. The bulk density of the original fresh chips was not assessed, therefore the effect of starting moisture content has not been accounted for here.

When comparing the chips created when chipping freshly harvested rods to those created after storage of rods, the proportion of finer particles, smaller than <3.15 mm, almost doubled after storage (Table 2). There was a corresponding 6% and 8% decrease in the 3.15–16 mm and in larger, 16–45 mm particle fractions, but these changes were not statistically significant (p > 0.05, t-tests). Also, there was no significant difference (p = 0.394, *t*-test) in the bulk density of the chips before and after storage (average 181 kg/m^3^). It was noted that a number of fines were present during the chipping process but, as this process took place outside, it was not possible to collect or quantify them. The only estimate of processing losses was made by comparing the weight of rods and weight of chip collected in 2014. In this case, the stored rod stack weighed 672 kg DM and, following chipping, it weighed 634 kg DM. Assuming no change of moisture content during chipping, this suggests that the DM loss during processing of the rods was around 5.7%.

## 4 Discussion

### 4.1 Dry matter losses and temperature profiles

The results from our study indicate that storing willow as wood chips in stacks leads to higher DM losses than storing it as rods in stacks. A key limitation of this study was that the rod piles (1.5 × 1.5 × 1.5 m, or 0.8 tonnes) were smaller in size than the wood chip pile, and were also smaller than what would conventionally be produced using a rod harvester (e.g. 4 tonnes with a Stemster III), though some methods of rod storage involve creating windrows instead of piles [21]. Producing larger piles of rods may affect the ventilation within the stack, which could affect the DM losses. A number of studies suggest that storing biomass in larger forms helps to avoid DM losses, though few have examined willow rods. A few studies have compared various options for harvesting poplar in Italy, and few have managed to get consistent sizes of piles; for example, Pari et al. [7], found that rod piles with a dimension of 3 × 3 × 2 m, showed a DM loss of 8.5%, though a larger pile (12 × 8 × 4 m) of poplar chips showed a DM loss of 10%. Generally they found that poplar rods dried more effectively than a range of (e.g. covered, uncovered) chip piles, and showed similar temperatures to ambient during the course of the experiment [22]. A more recent study [21], which laid cut poplar trees in windrows rather than discrete piles, found virtually zero DM losses. Therefore there is evidence that ventilation may affect the dry matter losses from rod storage and that pile size is important.

The whole-stack DM loss for wood chips was comparable to the previous year's experiment with SRC willow stored on concrete, which showed a total stack DM loss of 21% over three months, and average DM losses of 19% in plastic mesh bag samples [2]. That study also reported average DM losses of 18% from bags in a grassland-based stack, where it was not possible to weigh the entire stack to determine a whole-stack loss. The current results are consistent with trials using poplar stacks, covered in fleece and stored on concrete [3]. Poplar is similar to willow in the way that it is managed to produce relatively small stems, and the biomass will contain relatively high quantities of bark. It is important to note here that wood chip storage studies performed in locations with warmer climates generally show a lower DM loss during storage, for example two studies performed in Italy on poplar chips storage showed DM losses of around 5.1–9.8% for covered and uncovered piles that were 6 × 2.5 and 2 m in dimension [23], and the aforementioned larger pile in Ref. [7] showed a loss of 10% DM.

A higher DM loss with wood chip storage may simply be attributed to a higher surface area of material in chip which exposes readily available sugars and starches to microbial degradation [24], and the comminution process is believed to trigger a wound response in the cells of the plant which are still living [1]. This could also explain why the self-heating (mesophilic) phase seen in the wood chip stack was absent in the rod stacks. Degradable components may only represent a relatively small proportion of the biomass [25], so the self-heating phase only lasted for four days, after which the stack cooled to mesophilic temperatures, indicating a shift towards the decay of more recalcitrant compounds such as cellulose, hemicellulose and lignin, which are mainly decomposed by slow-acting fungi that operate best at lower temperatures. Lenz et al. (2015) suggested that mesophilic fungi are responsible for the majority of dry matter losses in wood storage. They found that dry matter losses in poplar chips accelerated as the stack cooled to around mesophilic temperatures (around 30 °C), about 2–3 months after stack establishment.

Another indicator of increased decomposition in the wood chip stack was the observed decrease in the C:N ratio in the stored chip, which was not observed in the rod samples. This is a common effect after the composting of biodegradable material due to the relative consumption of C and N during microbial decomposition [12]. This further supports the theory that biodegradable carbohydrate was being degraded during storage, despite there not being a clear change in ash contents.

The losses observed in the rod piles were more uncertain, mainly due to the higher range in the MC of the original rods, which was unexpected in comparison to the relatively constant MCs of the fresh wood chips. From the data, a total of approximately 10% DM loss was estimated, based on the weights of the whole stacks, those of the independent bundles and processing losses. A lack of self-heating suggests that the stacks were well ventilated, and that this, rather than the self-heating processes, was responsible for the effective drying seen. There was little evidence of change in composition due to storage, suggesting that little decomposition had taken place. The observed DM losses could be due to defoliation or breakage of smaller branches.

The results of the study therefore suggest that microbial decomposition is the main determinant of DM losses in wood chip storage, rather than handling losses. In both this study and a previous experiment [2] it was found that the DM losses from plastic mesh bags was in proportion to the whole stack-loss, suggesting that the DM losses due to handling and loss to soil were probably small, or 1–2% of the original DM and less than those from handling and processing rods (>5%). It is possible that the handling losses from wood chips would be higher if smaller wood chip piles were used, though in practice, it is recommended to have a larger heap to increase the ratio of core to crust [2], and this also requires a smaller surface area to store an equivalent amount of chip. It was previously suggested, from a study in Sweden, that smaller wood chip piles behave differently to larger ones, showing less rapid temperature increases, poorer rates of natural drying, and lower overall DM losses [5,17]. A more recent study in Italy showed that small (∼11t) and “medium” (∼23t) sized poplar chip heaps both showed a DM loss of 10% after being stored for four months, though it is not stated what the piles were built on, nor whether there was a loss of chip at the bottom of the heap due to soil contamination [26]. This reiterates the importance of location and climate when interpreting the results of wood chip storage studies; a factor which is becoming increasingly evident in the literature [2,23,26].

### 4.2 Quality changes

Storing willow as rods in small piles produces a drier and less variable chip than storing it as wood chip in stacks. In this experiment, the rod chips would be classified as A2 quality under the International Standards Organisation (ISO) 17,225–4 [27]. This is despite the freshly harvested rods having a more variable moisture content than the chips, probably due to the fact that the crop had started to produce new leaves by the time it was harvested (30^th^ March). In the wood chip storage stack, although the core dried quite well (from 56.4% to 38.2%) and was relatively consistent, it still would be classified as a lower quality chip (B1). Also, the lifting and mixing of the wetter apex and crust of the stack lead to a net higher MC (Fig. 3). In this case the moisture content limit would depend on the fuel specifications of the combustion technology used by the final consumer of the biomass.

In biomass, ash is derived from the minerals that the growing plant has incorporated during its lifetime and from material that originates from contamination during handling [28]. In this study, the differences in ash contents pre- and post-storage were not substantial, but this has also been observed in other studies [29]. As ash is a relatively small component of biomass, a great number of samples are required to capture the size of differences precisely between treatments. Overall, no correlation between DM losses and ash content could be found. Variation in ash content can be due to differing bark content over samples [3,30], which is difficult to control given the nature of coppiced plants having many small stems. Also, the mixed wood chip stack samples may have incurred some soil contamination during stack lifting. This will be a risk when storing wood chips on grassland, and consequently it is generally recommended that chips are stored on paved areas [31].

After storage, the rod chips had a higher lower heating value (LHV) and showed lower DM losses from storage, but they also contained a larger proportion of fines compared to the stored wood chips. Indeed, the dried rods clearly presented a more brittle material than fresh rods. A high fine content can lead to additional losses from dispersal of material to the air if the rods are not chipped in a controlled environment. During transport and distribution this can lead to a build-up of dust on surfaces that can heighten explosion and fire risks, as well as there being occupational health hazards associated with fines [15]. Therefore, additional losses from post-handling of rod material must be considered in the economic assessment of a rod supply chain.

## 5 Conclusion

Storing SRC willow in small heaps of rods leads to lower DM losses than storing as chip and produces a higher quality fuel in terms of moisture content and ash content, but has a problem of producing extra fines. Differing temperature profiles between the experimental stacks suggest that there is heightened microbial activity in wood chip stacks as opposed to stacks of rods. Comparison between discrete samples of chips and rods within the stacks and at the whole-stack level suggests that DM losses are primarily due to microbial and fungal decay rather than handling. The study highlights the challenges when producing a quality biomass fuel from willow while maximising the net yield. As the fine content of the chips did not change during storage it may be the case that forced drying and then storage of dried chip is the most viable option for producing the best quality fuel, though there will be trade-offs of increased costs and GHG emissions. More experimental work is needed to test the DM losses of larger piles of rods as this could affect ventilation within the piles and therefore affect their behaviour during storage.
